# How generative AI voice agents will transform medicine

**DOI:** 10.1038/s41746-025-01776-y

**Published:** 2025-06-12

**Authors:** Scott J. Adams, Julián N. Acosta, Pranav Rajpurkar

**Affiliations:** 1 Department of Medical Imaging, Royal University Hospital, University of Saskatchewan, Saskatoon, SK Canada; 2 Department of Biomedical Informatics, Harvard Medical School, Boston, MA USA

**Keywords:** Health services, Health care

## Abstract

Generative AI voice agents—conversational systems powered by large language models that can understand and produce natural speech in real time—are poised to transform how health systems engage with patients. While technical and implementation challenges remain, with thoughtful design, rigorous validation, and responsible deployment, generative AI voice agents could become a critical extension of the care team, increasing the reach of clinicians and health systems in ways previously limited by human resources.

Communication is the foundation of medicine. From diagnosis to shared decision-making, effective dialog between patients and clinicians drives outcomes^1^. Yet in a health system increasingly burdened by time constraints, staffing shortages, and growing administrative demands, sustaining high-quality, personalized communication with patients at scale has become increasingly difficult^2^. Generative AI voice agents offer a new solution. Generative AI voice agents are conversational systems powered by large language models that can understand and produce natural speech in real time, enabling dynamic, context-sensitive interactions with patients.

Unlike traditional chatbots which typically rely on pre-coded workflows that move from one question to the next with utility predominantly for only narrow tasks^3,4^, generative AI voice agents draw on large language models that have been trained on extensive corpuses of medical literature, anonymized patient transcripts, and other relevant datasets. This enables them to generate unique responses based on context and the specific details of a patient’s query or response, engaging with patients using natural speech. Rather than selecting from a set of predetermined prompts, generative AI voice agents can produce new sentences and tailor their messaging in a way that reflects individual patients’ concerns. Generative AI voice agents have the potential to pause to clarify incomplete or contradictory statements, detect nuances in how patients describe symptoms, and integrate multiple data points—when they are given access to them—from health records or previous calls. The generative component allows these voice agents to better handle unexpected questions and clinical nuances that often arise during medical conversations, all while relying on natural speech between the patient and the agent. As illustrated in Fig. 1, generative AI voice agents can integrate prior data and dynamic task lists to guide personalized interactions with patients.

**Fig. 1:**
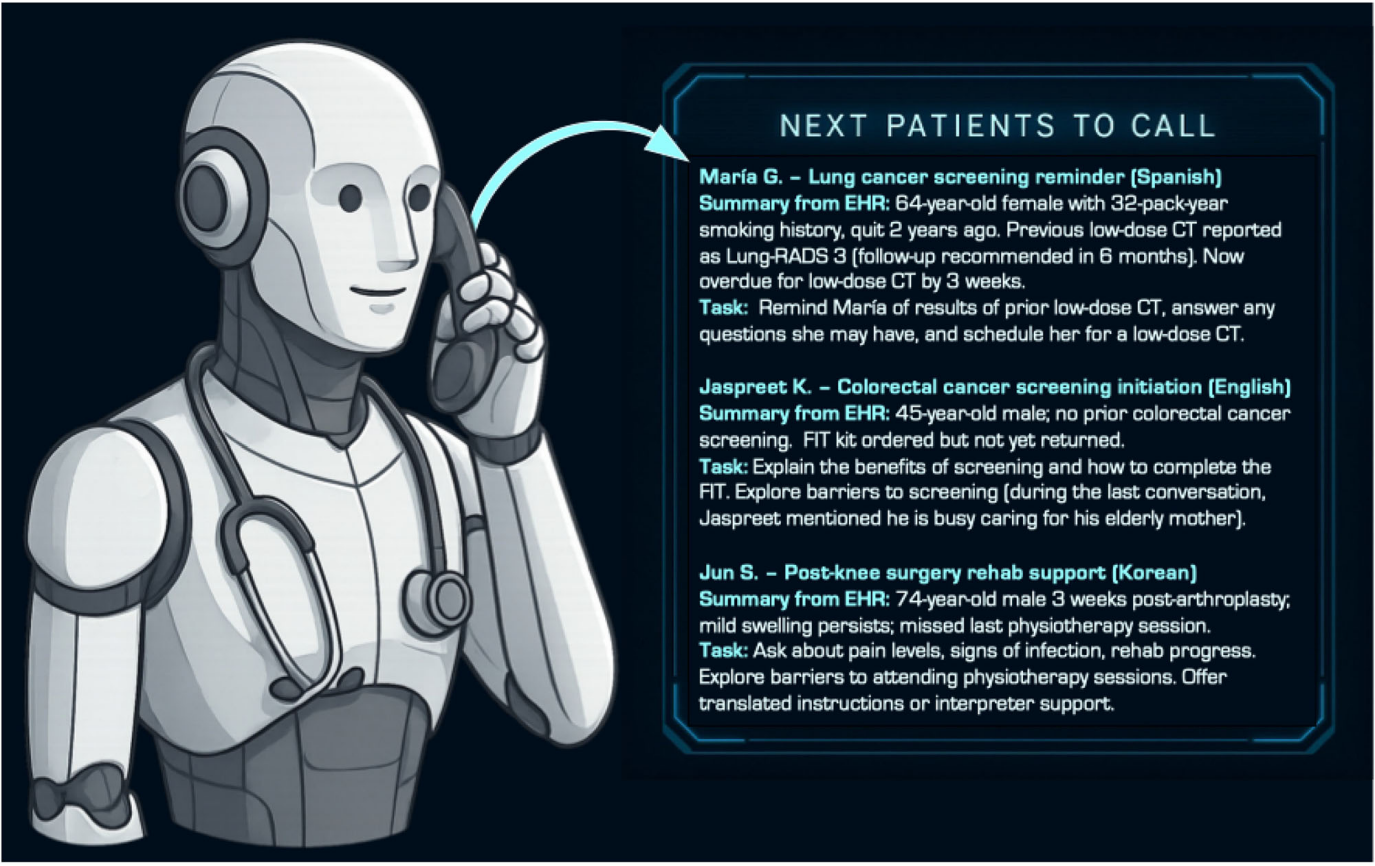
Generative AI voice agent engaging in a patient phone call while referencing a dynamic call list. For each patient, a large language model summarizes key information from the electronic health record and prior conversations and determines required tasks, which the generative AI voice agent then uses to guide real-time conversations with patients.

The new era of generative AI voice agents will allow us to substantially rethink use cases for what have been previously thought of as chatbots. These agents can triage symptoms, perform daily check-ins for chronic disease management, track medication adherence, and escalate concerns to clinicians, for example. With regular check-ins, agents can detect early signs of clinical deterioration, such as shifts in symptom descriptions or mood, enabling timely intervention before crises occur. One large-scale safety evaluation involving over 307,000 simulated patient interactions—each reviewed by licensed clinicians—suggested that generative voice agents can achieve medical advice accuracy rates exceeding 99%, with no instances of potentially severe harm^5^. However, findings should be interpreted as preliminary as the study is a preprint and not yet peer-reviewed. These results suggest that with appropriate oversight, generative AI voice agents can become trusted partners in delivering high-quality, patient-centered care. On the administrative side, these agents can handle billing questions, insurance verification, appointment reminders, and rescheduling. They can minimize the travel burden for patients with mobility limitations or geographic barriers to care by identifying opportunities for virtual appointments, clustering multiple appointments on the same day, and assisting with transportation arrangements. In addition, AI voice agents can serve as navigators for patients with complex medical needs or limited health literacy. They can enhance pre-appointment education and facilitate more meaningful clinical encounters by increasing patient preparedness. Although not Food and Drug Administration (FDA) cleared, several generative AI voice agents are already commercially available from companies such as Hippocratic AI^6^, Hyro^7^, and Orbita^8^. These companies have developed AI voice agents which facilitate basic tasks such as automated appointment scheduling and prescription refills, as well as tasks requiring more complex and personalized interactions such as symptom triage, chronic disease monitoring, and medication adherence support. Pair Team, a technology-enabled medical group that works with high-need Medicaid beneficiaries in California, built an AI agent to call physicians’ offices to schedule appointments, reducing the time that community health workers dedicate to these tasks and enabling them to spend more time building relationships with patients.

Most importantly, generative AI voice agents offer new opportunities to improve population health outcomes that were previously not possible at scale due to resource constraints. For the first time, health systems can offer proactive, personalized outreach to entire populations, boosting uptake of preventive services by reaching out with tailored reminders for cancer screening, vaccinations, or follow-up appointments, adapting language and tone based on a patient’s health literacy or cultural background. For example, a recent study evaluated a multilingual generative AI voice agent deployed to improve colorectal cancer screening rates among underserved populations. The AI agent conducted personalized, language-concordant outreach calls and achieved higher engagement among Spanish-speaking patients compared to English speakers—including more than double the fecal immunochemical test (FIT) test opt-in rate (18.2% vs. 7.1%) and longer call durations (6.05 vs. 4.03 min)—suggesting that, when thoughtfully designed and deployed, AI voice agents can reduce disparities in traditionally underserved populations and enhance preventive care engagement^9^. During extreme weather events, agents can monitor at-risk seniors for heat-related symptoms, remind them to stay hydrated, and ensure they have access to medications and support services. They can enable rapid population-wide response in emergencies, such as during a pandemic, where AI agents can screen for symptoms and provide isolation guidance, or during wildfire seasons, where they can identify patients with respiratory conditions, advise on air quality precautions, and provide real-time guidance without needing surge staffing.

However, widespread implementation of generative AI voice agents remains limited by several technical and operational hurdles. One technical hurdle is latency: generative AI models, especially those that rely on large-scale transformers, can be computationally intensive, leading to awkward pauses that break the illusion of human-like conversation^10^. Optimizing model performance, hardware, and cloud infrastructure will be critical to delivering seamless, real-time dialog. Another significant challenge is accurate turn detection, the process of determining when a patient has finished speaking so that the agent can respond appropriately. Turn detection typically employs voice activity detection, yet current implementations remain fragile and susceptible to errors, resulting in premature interruptions, awkward gaps in the conversation, or misunderstandings. Enhancing the reliability of turn detection mechanisms—such as by integrating semantic understanding, prosodic features, and contextual inference—will be essential to supporting fluid and high-quality conversations with generative AI voice agents in patient-facing applications.

More critically, there are safety risks. Whenever a generative AI voice agent delivers advice in a medical context, particularly over voice calls that might seem highly authentic, there is a danger that patients will treat the guidance as definitive. If the AI suggests self-care for a patient who is actually experiencing a life-threatening issue, the consequences can be catastrophic. To safeguard against this, generative AI voice agents must be built with robust clinical safety mechanisms. Urgent life-threatening scenarios—or cases in which the generative AI agent is uncertain—should trigger automatic escalation to a clinician. This requires models trained on domain-specific conversational data to recognize key symptoms, phrases, or emotional cues that indicate urgency, as well as mechanisms to monitor the AI model’s own level of uncertainty, to appropriately route such cases for review.

To clarify appropriate applications and associated risks, a tiered framework can help guide deployment and oversight of generative AI voice agents. Low-risk tasks include administrative functions such as scheduling or billing; moderate-risk tasks include preventive outreach and reminders; and high-risk tasks involve medical advice, triage, or clinical decision support. To support patient safety, many AI voice agents will be required to conform to medical device regulations, which continue to evolve. A generative AI voice agent would currently be classified as Software as a Medical Device (SaMD) when it performs functions intended for medical purposes, such as diagnosing, monitoring, or treating disease^11^. Voice agents built on locked models with fixed parameters and outputs may align more readily with existing regulatory pathways. In contrast, adaptive or open-ended models—whose behavior may shift over time due to updates in contextual data—pose challenges for traceability, reproducibility, and the potential need for ongoing validation. Liability also remains a gray area: responsibility for harm may be shared or contested among developers, clinicians, and health systems^12^.

Limited clinical validation compounds current challenges towards the implementation of generative AI voice agents. Prospective studies tailored to the agent’s intended use and associated level of clinical risk are essential to evaluate the effectiveness and safety of AI voice agents. For high-risk applications—such as those that provide therapeutic recommendations—randomized controlled trials will be necessary to assess clinical outcomes and patient safety risks. In contrast, for lower-risk functions such as administrative support or general health education, pragmatic evaluations, usability testing, and implementation studies may offer more efficient and contextual insights. Monitoring patient safety risks will require a combination of pre-deployment strategies, including simulation studies and prospective cohort studies, and post-market surveillance. Assessment of AI voice agents across diverse populations will be critical to assess model bias. While one large-scale study has shown the safety of generative AI voice agents^5^, until large-scale prospective studies or randomized controlled trials demonstrate that generative AI voice agents improve outcomes and reduce costs (or at least do not cause harm), many clinicians will hesitate to rely on them for anything beyond administrative assistance.

User-facing design considerations are critical to ensure the effectiveness of generative AI voice agents^13^. Despite the advantages of patients being able to interact with AI agents through voice, the next generation of agents should support multiple modes of delivery—including not only phone calls, but also video calls and text-based messaging—and adapt the modality to the user’s context, clinical scenario, and preference. For example, phone-based interfaces may be preferable for users without internet access, while text-based systems might support users who do not have a private environment for a phone conversation. Video calls may support the development of patient rapport, but this has yet to be studied in the context of generative AI agents. AI agents must accommodate patients with sensory impairments, such as by offering real-time speech-to-text transcripts for those with hearing loss or alternative input methods for those with speech difficulties, and be intuitive for individuals with limited digital literacy. Inclusive and adaptable design approaches will support usability across diverse patient populations.

Public trust in generative AI voice agents is essential for sustained adoption. Patients may be skeptical of these technologies due to prior experiences with spam calls, robocalls, and poorly functioning chatbots. Even if trust is initially established, engagement may erode over time, much like alert fatigue observed with early warning systems. Cultural attitudes, perceived impersonality, or fear of depersonalized care can further exacerbate disengagement. Incorporating elements of personalization—such as remembering prior interactions, using language aligned with a patient’s cultural context, and demonstrating empathy—can help establish these agents as legitimate members of the care team. Demonstrating responsiveness, reliability, and competence will be critical for AI voice agents to maintain patients’ trust and long-term engagement. Recent studies suggest that generative AI systems in healthcare can meet these challenges. For example, a study of a large language model-based AI system optimized for clinical history-taking and diagnostic dialog showed higher ratings than primary care providers in multiple domains, including eliciting information, explaining relevant clinical information, and managing patient concerns, although the system remains only text-based^14^.

For health systems, the successful adoption of generative AI voice agents depends not only on choosing the right tools, but on preparing the workforce to use them effectively. Clinicians, nurses, front-desk staff, and care coordinators will need to understand how these agents function, when to intervene, and how to override the system if necessary. This may require hiring or retraining staff for emerging roles in AI oversight, including individuals capable of interpreting AI outputs and recognizing system limitations. Concerns about AI replacing human labor often miss a more urgent reality: healthcare operates in a chronically supply-constrained environment with a shortage of clinicians and other healthcare workers. Generative AI voice agents will increasingly serve as a first line of engagement, handling some tasks such as routine follow-ups autonomously, while collaborating with clinicians on more complex or high-risk scenarios through defined escalation pathways.

Health system administrators must weigh the cost of acquiring or licensing advanced AI technology, integrating it with EMRs, training staff, and ensuring ongoing maintenance against the potential benefits in patient outcomes, operational efficiency, and bottom-line finances. Measuring these outcomes requires a holistic view. Do patients with chronic conditions show higher medication adherence and fewer complications when supported by generative AI voice agents? Do generative AI voice agents reduce emergency department visits or hospital readmissions? Do generative AI voice agents improve operational efficiency, such as reducing administrative workload or call center volume? If an organization can link generative AI voice agents to improved outcomes, higher reimbursements, or lower costs, the case for investing in generative AI voice agents becomes more compelling.

Generative AI voice agents are poised to transform how health systems engage with patients, expanding the reach of personalized, responsive communication in ways previously limited by human resources. While technical and implementation challenges remain, with thoughtful design, rigorous validation, and responsible deployment, generative AI voice agents could become a critical extension of the care team, supporting clinicians in delivering more equitable, efficient, and scalable healthcare.

## Data Availability

No datasets were generated or analyzed during the current study.
